# Impairment of Working Memory, Decision-making, and Executive Function in the First-Degree Relatives of People with Panic Disorder: A Pilot Study

**DOI:** 10.3389/fpsyt.2017.00219

**Published:** 2017-11-03

**Authors:** Zhenhe Zhou, Dongjie Ni

**Affiliations:** ^1^Department of Psychiatry, Wuxi Mental Health Center of Nanjing Medical University, Wuxi, China; ^2^Grade 2013 Class 3, Basic Medicine College of Liaoning Medical University, Jinzhou, China

**Keywords:** panic disorder, first-degree relatives, working memory, decision-making, executive function

## Abstract

**Background:**

Panic disorder (PD) patients present impairments of working memory, decision-making, and executive function. However, whether the first-degree relatives (FDRs) of people with PD present abnormal characteristics, including clinical and neuropsychological aspects, in comparison to the general population, has not been studied. Investigation and understanding of the abnormal neuropsychological characteristics of the FDRs of people with PD will contribute to the prevention and treatment of PD.

**Objective:**

The purpose of this paper is to compare the working memory, decision-making, and executive function among people with PD, their FDRs, and controls.

**Materials and methods:**

Neuropsychological functions of 30 people with PD, 30 FDRs of people with PD, and 30 controls were measured with a digit span task, Iowa Gambling Task (IGT), and Wisconsin Card Sorting Test (WCST).

**Results:**

Perseverative errors, failure to maintain set scores, and number of cards chosen from decks A, B, C, and D were higher for People with PD and their FDRs than those of controls. Furthermore, error rates for these tests were higher for people with PD than their FDRs. Forward scores and backward scores, percentage of conceptual level responses, the number of categories completed, choices from advantageous minus disadvantageous decks, and mean amount of money earned of people with PD and their FDRs were all lower than those of controls. Scores for these tests were also lower for people with PD than for their FDRs.

**Conclusion:**

People with PD as well as their FDRs present different degrees of impairments of working memory, decision-making, and executive function. Impaired performance on three tasks appears to be associated with the diathesis for PD and may be a valuable indicator of susceptibility for this disorder.

## Introduction

Panic disorder (PD) is a neurosis that presents as frequent panic attacks as well as obvious behavioral changes and leads to many dysfunctions in social function and professional capacity. According to the Diagnostic and Statistical Manual of Mental Disorders, Fifth Edition (DSM-5), PD is diagnosed as an anxiety disorder based primarily on the occurrence of panic attacks, which are persistent and often unexpected. Additionally, at least one attack is followed by the individual fearing that they will have more attacks for at least 1 month. This causes them to change their behavior, which often includes avoiding situations that might induce an attack (1). A common mental disorder, PD affects many people at some point in their lives. During the past three decades, many scholars living in different countries and geographic areas have suggested that the prevalence of PD has been increasing (2). Despite variability in prevalence, many studies have demonstrated consistently that PD is more common among females, in late adolescence or early adulthood, and when combined with depressive disorders and agoraphobia (3–5). For the pathogenesis of PD, many authors suggest that PD belongs to a disease of polygenic inheritance (6–8). With respect to lifetime prevalence, it has been reported that PD is 3–21 times higher in the first-degree relatives of people with PD in comparison to the general population (9). Twin studies showed that PD was heritable and that there is an increased genetic liability in patients with early onset PD (10, 11). In a study that investigated temperament in people with PD, their first-degree relatives and healthy individuals, the anxious, depressive, cyclothymiacs, and irritable temperament scores of people with PD were higher than those of healthy individuals, and no differences were reported between the people with PD and their relatives, with the exception of higher anxious temperament scores in people with PD (12). A previous study that investigated the effects of a 35% CO_2_ challenge test on healthy, first-degree relatives of people with PD and on healthy individuals indicated the first-degree relatives were more susceptible to the 35% CO_2_ challenge than the healthy individuals (13). Another study investigated anxiety sensitivity among people with PD, their first-degree relatives and controls found that the first-degree relatives were more anxiety-sensitive than the healthy controls, but less so than the people with PD (14).

Previous studies indicated that people with PD presented impaired information processing and attention as well as impaired interpretation of internal and external stimuli related to the disorder (15). Other studies also reported impairments in short-term memory in people with PD (16). Working memory mainly affects the capacity of brain executive function. A previous study has indicated that the prefrontal cortex is involved in human working memory (17). Additionally, it has been proven that people with PD present a dysfunction of frontolimbic circuits, in particular, orbitofrontal and cingulate cortices (18). Decision-making is an important cognitive function of the human brain. Many studies have reported that people with PD present with impaired decision-making. For example, one study suggested that enhanced cardiac perception may aid intuitive decision-making in healthy individuals, but impair intuitive decision-making in people with PD (19). That study, which used the two-choice prediction task to assess decision-making in people with PD, also showed that people with PD present abnormality of decision-making function (20). Executive functions are a capacity of the regulation or control for cognitive processes. The prefrontal areas of the frontal lobe are responsible to executive functions (21). A study indicated that people with PD with and without agoraphobia present dysfunctions in episodic memory (22). Another research study showed that the neurocognitive performance of PD and social phobia patients was lower than that of controls. Furthermore, people with PD exhibited deficits on short-delay free recall and verbal test performance in comparison to controls (23).

Summarily, people with PD present impairments of working memory, decision-making, and executive function. However, whether the first-degree relatives of people with PD present abnormal characteristics, including clinical and neuropsychological aspects, in comparison to the general population is still not reported. Investigation and understanding of abnormal characteristics of the first-degree relatives of people with PD, especially with regard to neuropsychological aspects, will contribute to the prevention and treatment of PD. Clinical psychiatrists and psychologists agree that the digit span task is useful to investigate verbal working memory, that the Iowa Gambling Task (IGT) simulates real-life decision-making, and that the Wisconsin Card Sorting Test (WCST) evaluates prefrontal executive functions. Until now, no studies using Digit Span Task, IGT and WCST in a comparison of working memory, decision-making, and executive functions among people with PD, their first-degree relatives, and controls have been reported.

The purpose of this study was to test whether the first-degree relatives of people with PD presented abnormalities of working memory, decision-making, and executive function compared with controls.

## Materials and Methods

### Time and Setting

We performed a cohort study. The experiment was completed in the Department of Psychiatry at Wuxi Mental Health Centre of Nanjing Medical University, China, from May 2013 to January 2015.

### Diagnostic Approaches and Participants

#### Study Population

##### PD Group

Inclusion criteria were as follows: (1) met the DSM-5 criteria for PD; (2) no medication was taken in the past 2 weeks; (3) age range from 18 to 65 years old; (4) were not smokers or alcohol dependent; (5) had never been diagnosed with substance dependence, neurological disorders, or history of head injury. The PD group was recruited from Wuxi Mental Health Centre. Thirty subjects were recruited in the PD group.

##### First-Degree Relatives of PD Patient Group

Participants in the first-degree relatives of people with PD (FDR-PD) group were selected from the first-degree relatives of patients in the PD group, with no more than one relative chosen for each PD patient, i.e., patient and relative pairs. Inclusion criteria were as follows: (1) no medication received in the past 2 weeks; (3) more than 18 years old; (4) were not smokers or alcohol dependent; (5) had never been diagnosed as substance dependence, neurological disorders or history of head injury. Thirty subjects were recruited as the FDR-PD group.

##### Controls

Thirty persons were selected for the control group, and their age, gender, and education level matched members of the FDR-PD group. Exclusion criteria were as follows: (1) smokers or alcohol dependent; (2) had been diagnosed with substance dependence or a neurological disorder or had a history of head injury; (3) had a family history of mental disorders. The controls were recruited from citizens living in Wuxi city, China through local advertisement.

At the start of the study, a psychiatric resident gathered clinical information about the subjects. The depressive and anxious symptoms of samples were assessed with the Hamilton Depression Scale (HAMD, version of 17 items) and Hamilton Anxiety Scale (HAMA).

All participants were given an informed consent form. All participants provided their own written informed consent to participate in this study and were paid $31.75. In this study, a clinician was required to judge whether all people with PD who participated in the study were able to understand the aims and risks of the study and to provide written informed consent. After the assessment, patients were recruited as subjects. The Ethics Committee of Wuxi Mental Health Centre of Nanjing Medical University, China approved the protocol for the research project.

##### Neuropsychological Tests and Procedures

The neuropsychological tests were as follows.

###### Digit Span Task

The Wechsler Adult Intelligence Scale-Revised China (WAIS-RC, computerized version), as described in detail previously (24), was used for measurement of the digit span task. The participant was told to listen because he or she will say a series of numbers and ask the participant to repeat them back in the same order. In this study, two main factors were used for analysis: ① forward scores; ② backward scores.

###### Iowa Gambling Task

The Iowa Gambling Task (IGT, computerized version), as recommended by Shurman et al. and as described in detail previously (25), was used to measure decision-making. Participants are told they may switch from deck to deck as often as they wish and that the overall goal of the task is to maximize profit on a loan of $2,000 of “play money.” After turning over each card, the subject receives an amount of money. However, on some cards, the subject receives money but also pays a penalty. In our study, choices from advantageous minus disadvantageous decks, mean amount of money earned, and number of cards chosen from decks A, B, C, and D were used for analysis.

###### Wisconsin Card Sorting Test

The WCST (computerized version VI), as described in detail previously (24), was used for measurement of executive function. The task entailed matching stimulus cards with one of four category cards in which the stimuli were multidimensional, according to color, shape, and number, with each dimension determining a sorting rule. In our study, five main types of WSCT were used for analysis: ① the total response errors; ② percentage of conceptual level responses; ③ perseverative errors; ④ the number of categories completed; ⑤ failure to maintain set.

##### Statistical Analysis

Data were calculated and analyzed using the Statistical Package for the Social Sciences (SPSS, Version 17.0, Inc. Chicago, IL, USA). The male to female sex ratios among members of the PD group, FDR-PD group and controls were analyzed with χ^2^ tests. A one-way analysis of variance (ANOVA) was used to compare age, years of education among PD group, FDR-PD group, and controls. HAMA and HAMD scores, digit span task scores, IGT scores, and WCST data were analyzed using one-way analysis of co-variance (ANCOVA) by setting age as the covariate. Bonferroni tests were adopted for pairwise comparisons.

## Results

### Demographic and Clinical Information of Participants

The demographic and clinical information of participants is given in the following Table 1.

**Table 1:** Demographic and clinical information of participants.

	PD	FDR-PD	Controls	Test statistic
Sex ratio (M/F)	30 (13/17)	30 (15/15)	30 (15/15)	χ^2^ = 6.52, *p* = 0.27, Φ = 0.19, NS
Mean age (SD)	50 (7)	26 (6)	26 (6)	*F* (2, 87) = 22.89, *p* = 0.021, Cohen’s *f* = 0.50; PD > FDR-PD and Controls; FDR-PD vs. Controls, NS
Age range	43–56	20–30	20–30	-
Education (SD)	9 (3)	9 (3)	9 (3)	*F* (2, 87) = 0.19, *p* = 0.73, Cohen’s *f* = 0.05; NS
HAMA (SD)	22.3 (4.0)	8.2 (3.1)	6.3 (3.4)	*F* (2, 87) = 43.81, *p* = 0.000, η^2^ = 0.510; PD > FDR-PD and Controls; FDR-PD vs. Controls, NS
HAMD (SD)	9.5 (4.0)	8.0 (3.6)	6.9 (3.2)	*F* (2, 87) = 56.84, *p* = 0.102, η^2^ = 0.460; NS

*PD, panic disorder patient group; FDR-PD, the first-degree relatives of people with PD group; M, male; F, female; HAMA, Hamilton Anxiety Scale; HAMD, Hamilton Depression Scale; NS, not significant*.

### Comparisons of Digit Span Task Scores among PD, FDR-PD, and Control Groups

A one-way ANCOVA for forward scores and backward scores revealed a significant main effect of group (PD, FDR-PD, and control groups). Forward and backward scores of the PD group and FDR-PD group were significantly lower than that of controls (for forward scores, *p* = 0.011 and 0.013; for backward scores, *p* = 0.010 and 0.028), while the above two factors of the PD group were significantly lower than those of the FDR-PD group (for forward scores, *p* = 0.025; for backward scores, *p* = 0.029) (Table 2).

**Table 2:** Digit Span scores: mean (SD) in PD group (*n* = 30), FDR-PD group (*n* = 30), and control group (*n* = 30).

Variable	PD	FDR-PD	Controls	Test statistic
Forward scores	8.5 (2. 1)	10.0 (1.8)	13.3 (1.8)	*F* (2, 87) = 42.22, *p* = 0.016, η^2^ = 0.40
Backward scores	8.3 (2.6)	10.1 (2.1)	12.4 (2.3)	*F* (2, 87) = 38.60, *p* = 0.023, η^2^ = 0.37

*PD, panic disorder patient group; FDR-PD, the first-degree relatives of people with PD group*.

### Comparisons of IGT Scores among PD Group, FDR-PD Group, and Controls

An one-way ANCOVA for choices from advantageous minus disadvantageous decks, mean amount of money earned, and number of cards chosen from decks A, B, C, and D showed a significant main effect of group (PD, FDR-PD, and control groups). Choices from advantageous minus disadvantageous decks and mean amount of money earned by the PD and FDR-PD groups were significantly lower than those of controls, and the number of cards chosen from decks A, B, C, and D by the PD and FDR-PD groups were significantly higher than those of controls (for choices from advantageous minus disadvantageous decks, *p* = 0.001 and 0.019; for mean amount of money earned, *p* = 0.045 and 0.049; for number of cards chosen from deck A, *p* = 0.020 and 0.033; for number of cards chosen from deck B, *p* = 0.043and 0.047; for number of cards chosen from deck C, *p* = 0.024 and 0.028; and for number of cards chosen from deck D *p* = 0.039 and 0.042).

Choices from advantageous minus disadvantageous decks and mean amount of money earned by the PD group were significantly lower than that of the FDR-PD group (*p* = 0.001 and 0.017), and the number of cards chosen from decks A, B, C, and D by the PD group were significantly higher than those of the FDR-PD group (*p* = 0.038, 0.039, 0.044, and 0.046) (Table 3).

**Table 3:** IGT scores: mean (SD) in PD group (*n* = 30), FDR-PD group (*n* = 30), and controls (*n* = 30).

Variable	PD	FDR-PD	Controls	Test statistic
Choices from advantageous minus disadvantageous decks	11.9 (10.3)	24.9 (12.0)	32.5 (14.1)	*F* (2, 87) = 41.10, *p* = 0.000, η^2^ = 0.56
Mean amount of money earned	1,913.1 (418.2)	2,115.3 (449.4)	2,211.5 (479.9)	*F* (2, 87) = 8.06, *p* = 0.031, η^2^ = 0.35
Number of cards chosen from deck A	19.8 (5.0)	18.0 (5.4)	15.6 (3.2)	*F* (2, 87) = 45.08, *p* = 0.039, η^2^ = 0.40
Number of cards chosen from deck B	25.6 (6.2)	22.0 (3.1)	19.3 (5.6)	*F* (2, 87) = 50.63, *p* = 0.028, η^2^ = 0.34
Number of cards chosen from deck C	25.3 (6.2)	28.0 (5.5)	32.1 (6.3)	*F* (2, 87) = 54.08, *p* = 0.015, η^2^ = 0.29
Number of cards chosen from deck D	28.1 (7.1)	29.0 (5.3)	32.7 (6.2)	*F* (2, 87) = 44.75, *p* = 0.013, η^2^ = 0.32

*PD: panic disorder patient group. FDR-PD: the first-degree relatives of People with PD group*.

### Comparisons of WCST Value among PD, FDR-PD, and Control Groups

An one-way ANCOVA for response errors, percentage of conceptual level responses, perseverative errors, the number of categories completed, and failure to maintain set showed a significant main effect of group (PD, FDR-PD, and control groups). Total response errors, perseverative errors, and failure to maintain set of the PD group and the FDR-PD group were significantly higher than that of controls, and the number of categories completed and percentage of conceptual level responses of the PD group and FDR-PD group were significantly lower than that of controls (total response errors, *p* = 0.022 and 0.017; perseverative errors, *p* = 0.030 and 0.035; failure to maintain set, *p* = 0.018 and 0.025; number of categories completed, *p* = 0.040 and 0.038; and percentage of conceptual level responses, *p* = 0.010 and 0.014).

Total response errors, perseverative errors, and failure to maintain set for PD group were significantly higher than for the FDR-PD group (*p* = 0.026, 0.029, and 0.021), and the number of categories completed and percentage of conceptual level responses of the PD group were significantly lower than that of the FDR-PD group (*p* = 0.037 and 0.043) (Table 4).

**Table 4:** WCST data: mean (SD) in PD group (*n* = 30), FDR-PD group (*n* = 30), and controls (*n* = 30).

Variable	PD	FDR-PD	Controls	Test statistic
Total response errors (%)	36.0 (11. 1)	25.3 (12.0)	17.9 (18.2)	*F* (2, 87) = 33.69, *p* = 0.001, η^2^ = 0.51
Conceptual level responses (%)	50.1 (7.4)	57.9 (7.5)	62.5 (8.0)	*F* (2, 87) = 26.12, *p* = 0.034, η^2^ = 0.30
Perseverative errors (%)	23.3 (11.6)	16.8 (12.1)	12.9 (10.1)	*F* (2, 87) = 53.18, *p* = 0.001, η^2^ = 0.41
Number of categories completed	5.6 (2.3)	6.2 (3.0)	6.7 (3.0)	*F* (2, 87) = 58.75, *p* = 0.021, η^2^ = 0.36
Failure to maintain set	0.8 (0.5)	0.6 (0.7)	0.4 (0.2)	*F* (2, 87) = 64.19, *p* = 0.015, η^2^ = 0.28

*PD, panic disorder patient group; FDR-PD, the first-degree relatives of people with PD group*.

## Discussion

This study tested working memory, decision-making and executive function among people with PD, their first-degree relatives, and controls. In the present study, working memory was tested with a digit span task, decision-making was measured with the Iowa Gambling Task, and executive function was assessed with the Wisconsin Card Sorting Test. Our results displayed working memory dysfunctions, decision-making impairments, and executive function deficiencies in people with PD and their first-degree relatives compared with controls.

Previous research suggested that working memory involves a system for the temporary storage and manipulation of information, forming a link between controlled action and perception (26). Many studies in animals and functional imaging of humans have identified the parietal cortex, frontal cortex, anterior cingulate, and parts of the basal ganglia involved in working memory (27–30). Our study results showed that two main functions of people with PD and their first-degree relatives were significantly lower than those of controls by the measurement of the digit span task, and forward scores and backward scores of people with PD were lower compared to their first-degree relatives. These results indicated that people with PD as well as their first-degree relatives all presented working memory dysfunction, with varying degrees of impairment between these two groups.

The Iowa Gambling Task is meant to simulate real-life decision-making processes and reflects the function of the orbitofrontal cortex (OFC) (25). In our study, test scores for choices from advantageous minus disadvantageous decks and mean amount of money earned by people with PD and their first-degree relatives were significantly lower than those of controls, and the number of cards chosen from decks A, B, C, and D by people with PD and their first-degree relatives were significantly higher than those of controls. Additionally, choices from advantageous minus disadvantageous decks and the mean amount of money earned by people with PD were significantly lower than those of their first-degree relatives, and the number of cards chosen from decks A, B, C, and D by people with PD were significantly higher than those of their first-degree relatives. Consistent with previous studies (22, 23), our results indicated that people with PD and their first-degree relatives presented different degrees of decision-making impairments.

Because the WCST is sensitive to frontal lobe dysfunctions, it is an assessment of executive process including strategic planning, organized searching, etc. (21, 31). Consistent with previous studies (22, 23, 32), our results showed that some factors of WCST for people with PD and their first-degree relatives were higher than those of controls, and other factors, such as the number of categories completed and the percentage of conceptual level responses, of people with PD and their first-degree relatives were lower than those of controls, which confirmed that people with PD as well as their first-degree relatives presented different degrees of executive dysfunction.

In our study, the first-degree relatives of people with PD exhibited impaired verbal working memory, decision-making, and executive function. These findings cannot be attributed to other factors such as lower education levels, medication effects, etc. Impaired performance on three tasks appears to be associated with the diathesis for PD and may be a valuable indicator of susceptibility for this disorder. Whether performance on the digit span task, IGT, and WCST may be used as an endophenotypic marker for the PD genetic diathesis should be researched in terms of other aspects in the future, such as neuroimaging, neurobiochemistry, and genetics.

## Conclusion

The first-degree relatives of people with PD presented abnormalities of working memory, decision-making, and executive function compared with controls.

The implication of our study is that understanding the abnormalities of the first-degree relatives of people with PD with respect to neuropsychological functions will contribute to the prevention and treatment of PD. A limitation of this study is that these results are preliminary due to the small sample size. It is necessary to replicate these findings with larger sample sizes in further studies.

## Ethics Statement

All participants were given a written informed consent and all were paid for$31.75, and all participants provided their own written informed consent to participate in this study. The Ethics Committee of Wuxi Mental Health Center of Nanjing Medical University, China approves the protocol for the research project.

## Author Contributions

ZZ and DN: conceived and designed the experiments, performed the experiments, analyzed the data, contributed reagents/materials/analysis tools, wrote the paper, read and approved the final manuscript.

## Conflict of Interest Statement

The authors declare that the research was conducted in the absence of any commercial or financial relationships that could be construed as a potential conflict of interest.
